# Fingolimod Prevents Blood-Brain Barrier Disruption Induced by the Sera from Patients with Multiple Sclerosis

**DOI:** 10.1371/journal.pone.0121488

**Published:** 2015-03-16

**Authors:** Hideaki Nishihara, Fumitaka Shimizu, Yasuteru Sano, Yukio Takeshita, Toshihiko Maeda, Masaaki Abe, Michiaki Koga, Takashi Kanda

**Affiliations:** Department of Neurology and Clinical Neuroscience, Yamaguchi University Graduate School of Medicine, Ube, Japan; Research Inst. of Environmental Med., Nagoya Univ., JAPAN

## Abstract

**Objective:**

Effect of fingolimod in multiple sclerosis (MS) is thought to involve the prevention of lymphocyte egress from lymphoid tissues, thereby reducing autoaggressive lymphocyte infiltration into the central nervous system across blood-brain barrier (BBB). However, brain microvascular endothelial cells (BMECs) represent a possible additional target for fingolimod in MS patients by directly repairing the function of BBB, as S1P receptors are also expressed by BMECs. In this study, we evaluated the effects of fingolimod on BMECs and clarified whether fingolimod-phosphate restores the BBB function after exposure to MS sera.

**Methods:**

Changes in tight junction proteins, adhesion molecules and transendothelial electrical resistance (TEER) in BMECs were evaluated following incubation in conditioned medium with or without fingolimod/fingolimod-phosphate. In addition, the effects of sera derived from MS patients, including those in the relapse phase of relapse-remitting (RR) MS, stable phase of RRMS and secondary progressive MS (SPMS), on the function of BBB in the presence of fingolimod-phosphate were assessed.

**Results:**

Incubation with fingolimod-phosphate increased the claudin-5 protein levels and TEER values in BMECs, although it did not change the amount of occludin, ICAM-1 or MelCAM proteins. Pretreatment with fingolimod-phosphate restored the changes in the claudin-5 and VCAM-1 protein/mRNA levels and TEER values in BMECs after exposure to MS sera.

**Conclusions:**

Pretreatment with fingolimod-phosphate prevents BBB disruption caused by both RRMS and SPMS sera via the upregulation of claudin-5 and downregulation of VCAM-1 in BMECs, suggesting that fingolimod-phosphate is capable of directly modifying the BBB. BMECs represent a possible therapeutic target for fingolimod in MS patients.

## Introduction

Fingolimod is a sphingosine-1 phosphate (S1P) receptor modulator (not only S1P1, but also S1P3, S1P4 and S1P5) approved as the first oral therapy for relapse-remitting (RR) multiple sclerosis (MS) and has been demonstrated to exhibit high efficacy in reducing the annual relapse rate in patients with RRMS [1]. Fingolimod is phosphorylated *in vivo* by sphingosine kinases to yield the active metabolite fingolimod-phosphate, which subsequently binds with S1P receptors, resulting in their internalization and degradation [2]. Fingolimod-phosphate acts as a functional antagonist to S1P1 receptors expressed on lymphocytes and prevents lymphocyte egress from lymphoid organs to the blood, thereby reducing autoaggressive lymphocyte infiltration into the central nervous system (CNS) [3–6]. In addition, recent evidence indicates that fingolimod may also have a direct effect on the S1P receptor expressed on various types of cells within the CNS, including astrocytes, oligodendrocytes, neurons and microglia [7]. However, the specific effects of fingolimod on brain microvascular endothelial cells (BMECs) comprising the blood-brain barrier (BBB) are not well understood, although a few reports have suggested that fingolimod-phosphate may also act on BMECs and modify the BBB function directly, as BMECs have been reported to express S1P_1_, S1P_2_, S1P_3_ and S1P_5_ receptors and type-2 SphK, which phosphorylates fingolimod into fingolimod-phosphate [8].

Pathological BBB breakdown includes two core factors: the paracellular leakage of soluble inflammatory mediators into the CNS via the disruption of tight junctions and the transcellular entry of inflammatory T cells across BMECs via the upregulation of adhesion molecules. Claudin-5 is recognized to be a key component of tight junction proteins, and the downregulation of this protein leads to an increase in the paracellular permeability of the BBB. The VCAM-1 present on BMECs is also an essential adhesion molecule, which plays a central role in the transmigration of T cells across the BBB. The blockade of VCAM-1 interactions prevents the binding of T cells to BMECs, eventually resulting in enhancement of the barrier properties of the BBB. We recently reported that sera derived from patients in the relapse phase of RRMS (RRMS-R) or secondary progressive MS (SPMS) decrease the claudin-5 protein levels and transendothelial electrical resistance (TEER) values in BMECs, while that derived from patients with RRMS-R, stable phase of RRMS (RRMS-S) and SPMS increases the VCAM-1 protein levels in BMECs [9]. In the present study, we examined the effects of fingolimod on BMECs and evaluated whether fingolimod-phosphate can be used to restore the function of the BBB after exposure to sera from MS patients.

## Materials and Methods

### Sera

This study was approved by the ethics committee of the Medical Faculty, Yamaguchi University, and written informed consent was obtained from each participant. This consent procedure was also approved by the ethics committee of Yamaguchi University. Serum was collected from nine MS patients diagnosed at Yamaguchi University Hospital. All patients met the clinical criteria based on the McDonald criteria [10]. In addition, serum was obtained within one week after the new appearance of symptoms from three patients in the relapse phase of RRMS (RRMS-R) who presented with both new worsening of neurological symptoms associated with objective neurologic signs and the appearance of new gadolinium-enhancing lesions on magnetic resonance imaging (MRI). Three patients with stable phase of RRMS (RRMS-S) who were being treated with IFN-β and had been in clinical remission for one year were also enrolled in this study. Furthermore, three SPMS patients who exhibited recently confirmed progression based on the Expanded Disability Status Scale (EDSS) score without relapse (EDSS>4.0) were enrolled. All patients (RRMS-R, RRMS-S and SPMS) included in this study were treated with IFN-β. Corticosteroids were not used when the blood samples were collected. None of the patients had a history of previous treatment with other disease-modifying therapies, including fingolimod, natalizumab or dimethyl fumarate. The blood samples were stored at −80°C until use. All sera were inactivated at 56°C for 30 minutes immediately prior to use.

### Reagents

The culture medium for the cells has been previously described [11]. Polyclonal anti-claudin-5 and anti-occludin antibodies were purchased from Zymed (San Francisco, CA, U.S.A). Polyclonal anti-actin, anti-beta tubulin, anti-p65 subunit of NFκB antibodies, monoclonal anti-intercellular adhesion molecule-1 (ICAM-1) and anti-melanoma cell-adhesion molecule (MelCAM) antibodies were obtained from Santa Cruz (Santa Cruz, CA, U.S.A). Polyclonal anti-vascular cell adhesion molecule-1 (VCAM-1) antibodies were purchased from R&D systems (Minneapolis, MN, U.S.A). Fingolimod and fingolimod-phosphate were provided by Mitsubishi Tanabe Pharma (Osaka, Japan).

### Cell culture and treatment with fingolimod or fingolimod-phosphate

Immortalized human BMECs were generated as previously described [11,12]. The cells were cultured in the conditioned medium with/without fingolimod or fingolimod-phosphate in a CO_2_ incubator at 37^○^C for 12 hours before total mRNA was extracted. The total protein was obtained, and the TEER value was measured 24 hours later.

### Treatment with fingolimod-phosphate and sera

The cells were pretreated with the conditioned medium containing 5 nM fingolimod-phosphate for 24 hours in a CO_2_ incubator at 37^○^C. The cells were cultured with the conditioned medium including 10% sera from the MS patients with or without fingolimod phosphate for 12 hours before total mRNA was extracted. The total protein was obtained, and the TEER value was measured 24 hours later.

### Western blot analysis

We used the same methodology as the one employed in a previous study [11]. The membranes were blocked and the blots were incubated with relevant primary antibodies (dilution 1:100) for one hour, followed by the secondary antibody (dilution 1:2,000) for one hour at room temperature. The bands were visualized by enhanced chemiluminescence detection system (ECL-prime, Amersham, UK). For quantification, each band density was corrected for the anti-actin or anti-beta tubulin band density using the Quantity One software program (Bio-Rad, Hercules, CA), and the changes in the expression of tight junction proteins, including claudin-5 and occludin, adhesion proteins, such as VCAM-1, ICAM-1 and MelCAM, and the p65 subunit of NFκB in the BMECs were examined.

### Quantitative real-time PCR analysis

For real-time RT-PCR, total RNA synthesized from the PBS-washed cells and single-stranded cDNA was prepared from 120 ng of total RNA. The samples were subjected to a PCR analysis, and the standard reaction curves were analyzed as previously described [13]. The sequences of the primers were as follows: forward primer (5’-CTGTTTCCATAGGCAGAGCG-3’) and reverse primer (5’-AAGCAGATTCTTAGCCTTCC-3’) for claudin-5 [14]; forward primer (5’-AAAAGCGGAGACAGGAGACA-3’) and reverse primer (5’-GCAAAATAGAGCACGAGAAGC-3’) for VCAM-1 [15]; forward primer (5’-ACTGTTCCCCCTCATCTTCC-3’) and reverse primer (5’-TGGTCCTGTGTAGCCATTGA-3’) for p65 subunit of NFκB [15]; forward primer (5’-GTCAACGGATTTGGTCTGTATT-3’), reverse primer (5’-AGTCTTCTGGGTGGCAGTGAT-3’) for glyceraldehyde-3-phosphate dehydrogenase (G3PDH) [16]. G3PDH was used as an internal standard. The Stratagene Mx3005P instrument (STRATAGENE, Cedar Greek, Texas, USA) was used to perform the quantitative real-time PCR analyses, and the relative quantity of each molecule was calculated according to the formula Rv = RGene/RGAPDH using a software program, as previously described [17].

### Transendothelial electrical resistance studies

The TEER values were measured using a Millicell electrical resistance apparatus (Endohm-6 and EVOM, World Precision Instruments, Sarasota, FL, U.S.A), as previously described [11] The effects of each type of medium (condition medium with or without fingolimod/fingolimod-phosphate, conditioned medium including 10% MS patient sera with or without 5 nM fingolimod-phosphate) were evaluated as the changes in the TEER values. The resistance of blank filters was subtracted from that of the filters with cells before the final resistance was calculated.

### Data analysis

All comparisons of the median values between the groups were made using the Mann-Whitney U test for continuous variables, and a two-sided p value of <0.05 was considered to be statistically significant.

## Results

### Treatment with fingolimod-phosphate increases the barrier properties of BMECs

**Fig 1:**
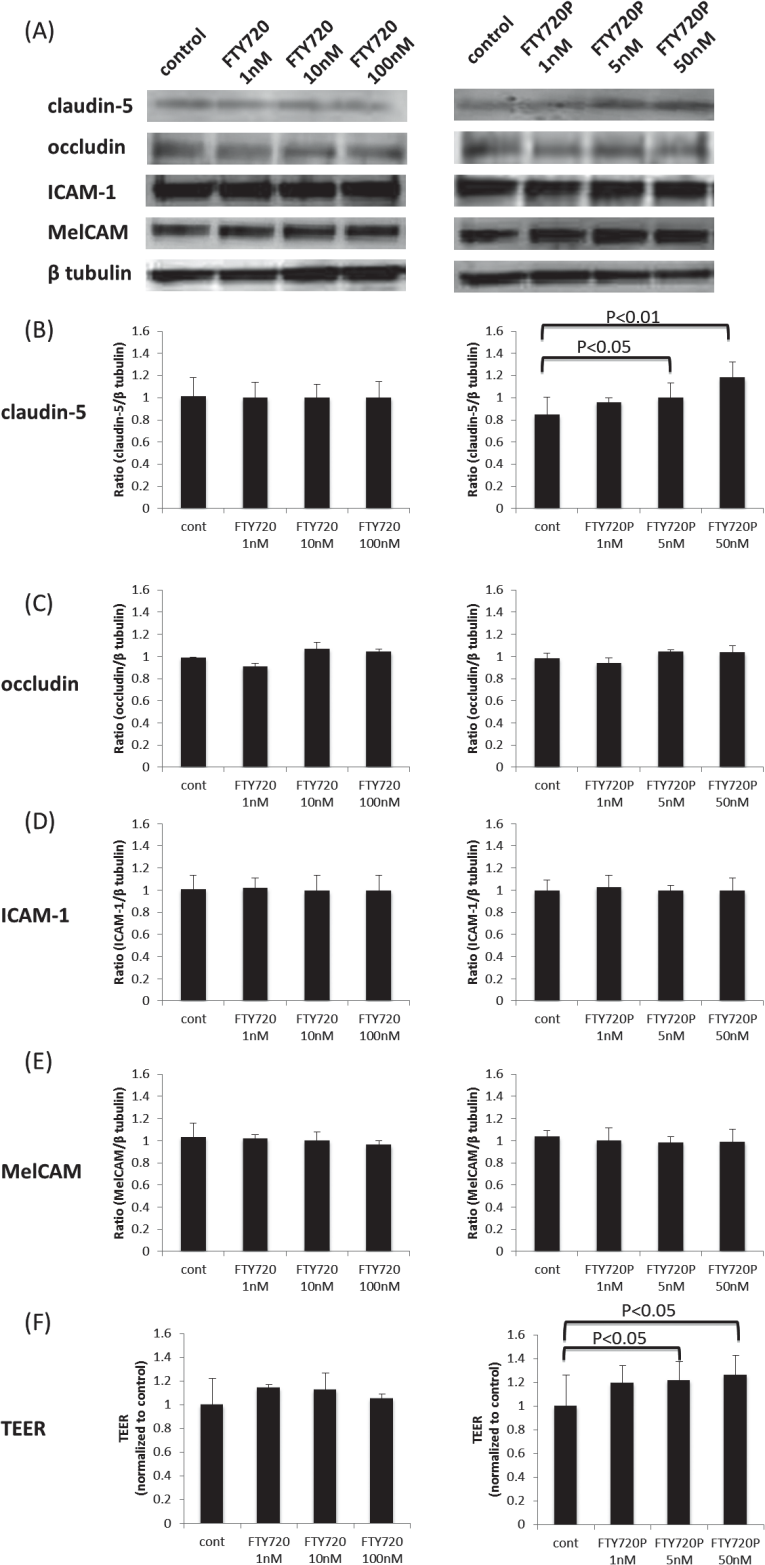
The effects of fingolimod and fingolimod-phosphate on BMECs. (A) The effects of fingolimod and fingolimod-phosphate on tight junction proteins, such as claudin-5 and occludin, and adhesion molecules, including ICAM-1 and MelCAM, in the human brain microvascular endothelial cells (BMECs) were measured using a Western blot analysis. (B)(C)(D)(E) Each bar graph reflects the combined densitometry data for the independent experiments. Treatment with fingolimod-phosphate increased the claudin-5 protein levels (B) and TEER values in the BMECs in a dose-dependent manner (F).

We examined the changes in the amount of tight junction proteins (claudin-5 and occludin) and adhesion molecules (ICAM-1, MelCAM and VCAM-1) after incubation with the conditioned medium with/without fingolimod or fingolimod-phosphate. Consequently, treatment with fingolimod-phosphate increased the claudin-5 protein levels (Fig. 1A-B) and enhanced the TEER values (Fig. 1F) in the BMECs, although it did not change the amount of occludin (Fig. 1A, C). On the other hand, incubation with fingolimod did not alter the tight junction protein levels or TEER values in the BMECs (Fig. 1A-C, F). Furthermore, the levels of adhesion molecules, including ICAM-1 and MelCAM, in the BMECs were not changed by treatment with either fingolimod or fingolimod-phosphate (Fig. 1A, D, E). The expression of VCAM-1 was not detected in the BMECs when the cells were incubated with conditioned medium that did not include the MS sera (data not shown).

### Pretreatment with fingolimod-phosphate prevents the barrier disruption caused by MS sera

**Fig 2:**
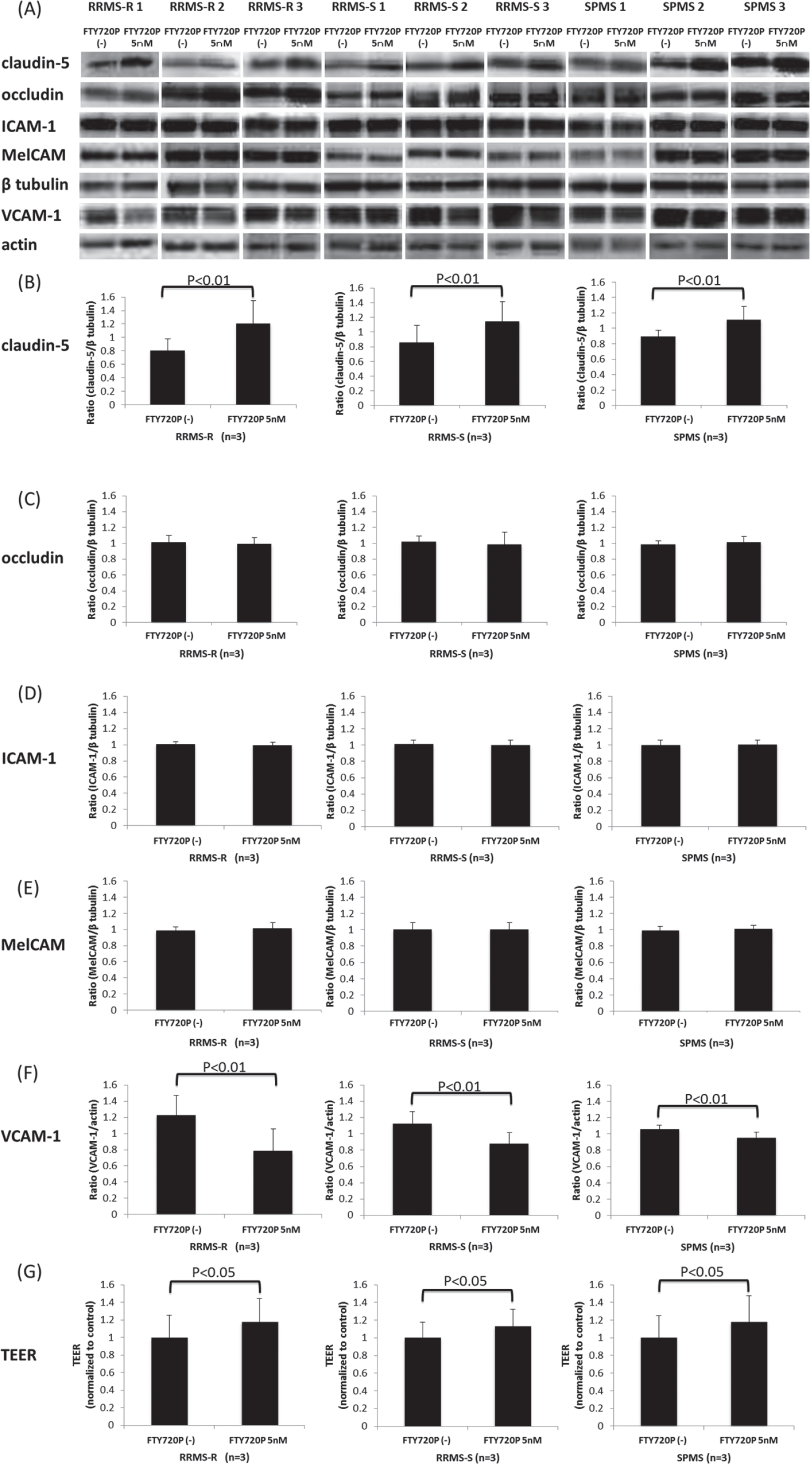
The effects of pretreatment with fingolimod-phosphate on BMECs in the presence of MS sera. (A) The effects of pretreatment with fingolimod-phosphate on the expression of tight junction proteins and adhesion molecules in the human brain microvascular endothelial cells (BMECs) in the presence of MS sera were determined using a Western blot analysis. (B)(C)(D)(E)(F) Each bar graph reflects the combined densitometry data for the independent experiments (mean ± SEM, RRMS-R n = 3, RRMS-S n = 3, SPMS n = 3). Pretreatment with fingolimod-phosphate resulted in an increase in the claudin-5 protein levels (B) and TEER values (G) and a decrease in the VCAM-1 protein levels (F) in the BMECs, which were upregulated after exposure to the sera from all MS patients, including the RRMS-R, RRMS-S and SPMS patients, compared to that observed in the cells not pretreated with fingolimod-phosphate.

Fig. 2 shows the effects of fingolimod-phosphate pretreatment following exposure to the sera of the nine MS patients, including three RRMS-R patients, three RRMS-S patients and three SPMS patients, on the expression of tight junction proteins (claudin-5 and occludin) and adhesion molecules (ICAM-1, MelCAM and VCAM-1) in the BMECs using a Western blot analysis. Pretreatment with fingolimod-phosphate significantly increased the claudin-5 protein levels and TEER values in the BMECs compared with that observed in the cells not pretreated with fingolimod-phosphate (Fig. 2A, B, G). In addition, pretreatment with fingolimod-phosphate resulted in a decrease in the VCAM-1 protein levels, which were upregulated after exposure to the sera of all MS patients with RRMS-R, RRMS-S and SPMS, compared with that observed in the cells not pretreated with fingolimod-phosphate (Fig. 2A, F). In contrast, the levels of occludin, ICAM-1 and MelCAM proteins in the BMECs were not influenced by fingolimod-phosphatate pretreatment (Fig. 2A, C-E).

### Pretreatment with fingolimod-phosphate reduces NFκB signals in MS patients

**Fig 3:**
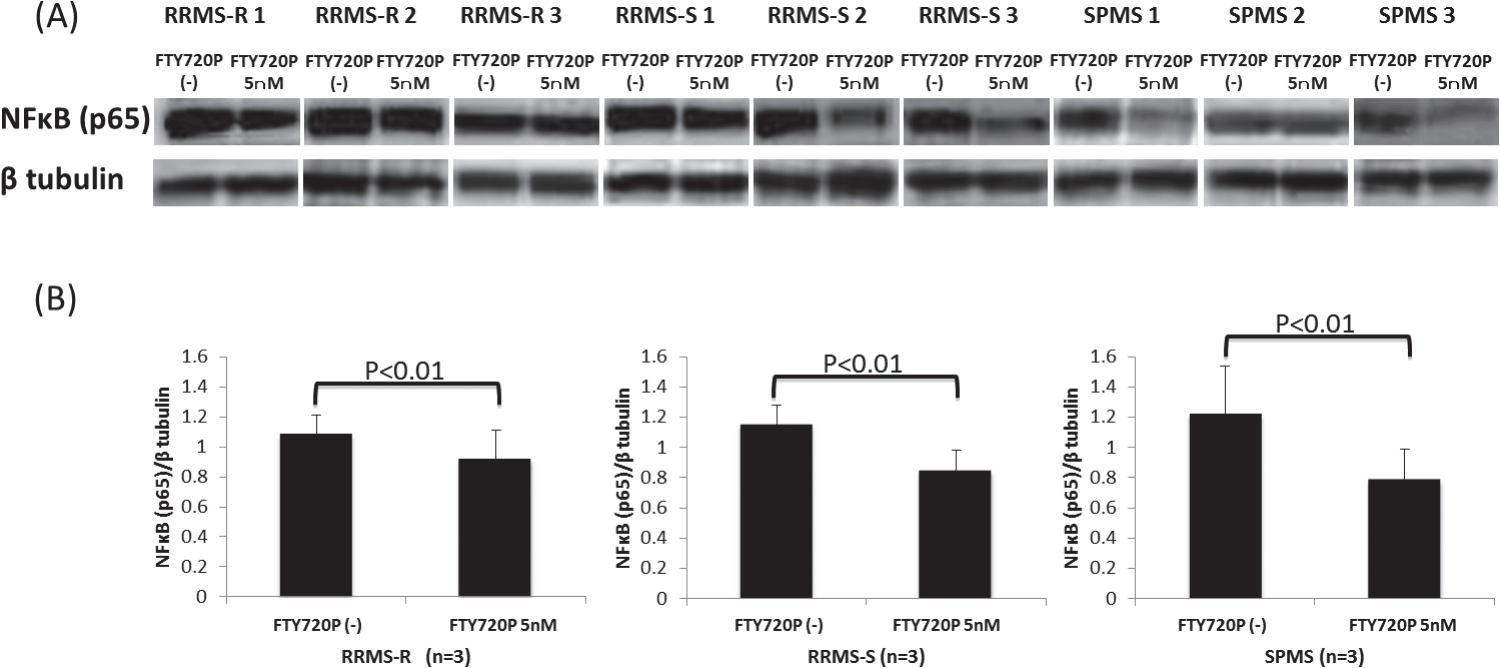
The effects of pretreatment with fingolimod-phosphate on NFκB in the presence of MS sera. (A) The effects of pretreatment with fingolimod-phosphate on the p65 subunit of NFκB in the human brain microvascular endothelial cells in the presence of MS sera were determined using a Western blot analysis. (B) Each bar graph reflects the combined densitometry data for the independent experiments (mean ± SEM, RRMS-R n = 3, RRMS-S n = 3, SPMS n = 3). Pretreatment with fingolimod-phosphate significantly decreased the protein levels of the p65 subunit of NFκB in the BMECs compared with that observed in the cells not pretreated with fingolimod-phosphate.

We next examined whether the downregulation of VCAM-1 proteins in BMECs is induced via NFκB signaling. Consequently, pretreatment with fingolimod-phosphate significantly decreased the protein levels of the p65 subunit of NFκB in the BMECs, which were upregulated after exposure to the sera from the RRMS-R, RRMS-S and SPMS patients, compared to that without treatment with fingolimod-phosphate (Fig. 3A-B).

### Pretreatment with fingolimod-phosphate influences the *claudin-5*, *VCAM-1* and *NFκB* mRNA expression

**Fig 4:**
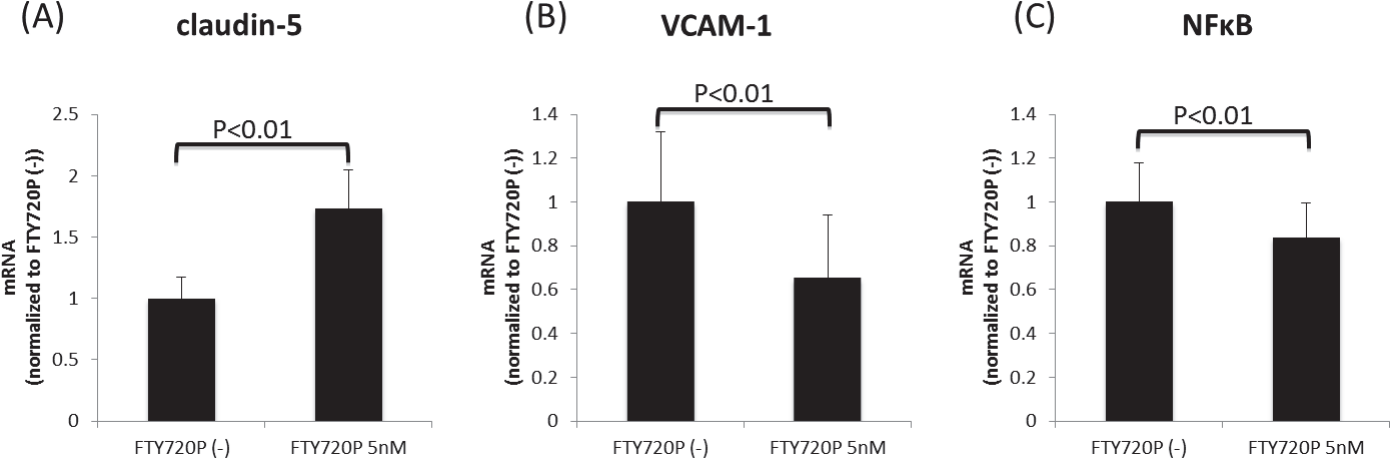
The effects of pretreatment with fingolimod-phosphate on the mRNA expression. The effects of pretreatment with fingolimod-phosphate on the mRNA expression of *claudin-5*, *VCAM-1* and *NFκB* in the human brain microvascular endothelial cells in the presence of MS sera were determined using a quantitative real-time PCR analysis. Pretreatment with fingolimod-phosphate resulted in the significant upregulation of the expression levels of *claudin-5* mRNA (A) and the downregulation of the expression levels of both *VCAM-1* and the p65 subunit of *NFκB* in the BMECs (B)(C) compared with that observed in the cells not pretreated with fingolimod-phosphate.

Using a quantitative real-time PCR analysis, we confirmed the effects of pretreatment with fingolimod-phosphate on the mRNA expression of *claudin-5*, *VCAM-1* and *NFκB* in the BMECs after MS sera exposure. Pretreatment with fingolimod-phosphate significantly increased the expression levels of *claudin-5* and decreased the mRNA levels of both *VCAM-1* and *NFκB* in the BMECs compared with that observed in the cells not pretreated with fingolimod-phosphate (Fig. 4A-C).

## Discussion

Fingolimod is thought to provide therapeutic effects in MS patients by preventing the egress of lymphocytes from lymph nodes, thus reducing the degree of infiltration of these cells in the CNS. However, in addition to their effects on lymphocytes, many recent studies have stressed that fingolimod also has a direct effect on several cells within the CNS, including astrocytes, oligodendrocytes, microglia and neurons [7,18]. As for the CNS vascular system, limited evidence has demonstrated that fingolimod acts on BMECs and modifies the BBB directly. For example, the integrity of the BBB is regulated by S1P receptors and fingolimod has been shown to make the BBB less permeable as well as decrease the ICAM-1 expression in BMECs via S1P1 signaling, particularly under inflammatory conditions [19–22]. In the present study, we showed that fingolimod-phosphatase increases the claudin-5 protein levels and TEER values in BMECs. This finding indicates that fingolimod has a direct effect on BMECs and subsequently enhances the BBB function. At the clinical dose for MS (0.5 mg/day), oral fingolimod undergoes rapid phosphorylation by sphingosine kinase 2 into fingolimod-phosphate *in vivo*, which is eventually present at a concentration of 4.7 nM in the blood. We believe that the concentration of 5.0 nM of fingolimod-phosphate incubated on the cells in the presence of MS sera in the present study is reasonable for examining the effects of circulating fingolimod-phosphate on the BBB in patients with MS.

Disruption of the BBB is considered to be a key step in the development of both RRMS and SPMS. Based on an extensive serial MRI study of patients with RRMS, the presence of gadolinium enhancement on MRI implying the breakdown of the BBB precedes the detection of clinical evidence of new lesions in RRMS patients [23]. The pathological findings of autopsy brain sections derived from patients with acute-phase RRMS or SPMS also show that the decreased expression of tight junction proteins is observed more frequently in active lesions than in inactive lesions and correlates with the leakage of serum fibrinogen, thus reflecting increased permeability of the BBB [24–27]. Furthermore, our previous data demonstrated that sera obtained from patients with relapsing RRMS as well as SPMS disrupt the BBB via a decrease in the expression of claudin-5 proteins in BMECs [9]. Taken together, these previous data suggest that it is important to identify candidate drugs capable of modifying the BBB in order to prevent relapse and disease progression in the setting of MS. In the present study, we first showed that fingolimod restores the BBB dysfunction induced by sera from MS patients; specifically, pretreatment with fingolimod-phosphate restored the decreased expression levels of claudin-5 protein/mRNA and TEER values in the BMECs following exposure to sera derived from RRMS and SPMS patients, thus suggesting that fingolimod-phosphate is able to restore BBB damage and make the BBB less permeable in both the relapse and progressive stages of MS.

VCAM-1 and VLA-4 have shown to be expressed at higher levels in MS lesions, although they are rarely detected in normal brain lesions [28]. As a ligand for the VLA-4 expressed on leukocytes, VCAM-1 plays an important role in transmigration across the BBB [29–32] The present findings demonstrate that pretreatment with fingolimod-phosphate decreases the expression levels of protein/mRNA in the p65 subunit of NFκB and VCAM-1, which were consequently upregulated after exposure to MS sera, thus suggesting the effect of this compound in decreasing the VCAM-1 expression in BMECs by inhibiting NFκB signaling. Although the beneficial effects of fingolimod are thought to be mediated via altered lymphocyte circulation, our data suggest that fingolimod-phosphate prevents the infiltration of autoaggressive lymphocytes into the CNS across the BBB via its peripheral actions in addition to direct effects on BMECs via the downregulation of VCAM-1. Natalizumab is a humanized monoclonal antibody that selectively targets the anti-α4 subunit of VLA4 and inhibits the interaction between VLA-4 and VCAM-1 during the transmigration of immune cells across the BBB [33]. The remarkable efficacy of this agent in preventing clinical and radiological relapse has not previously been documented using other disease-modifying strategies, although the use of this drug has been reported to be associated with the development of progressive multifocal leukoencephalopathy (PML). As the beneficial effects of fingolimod-phosphate against MS are, at least partially, due to the blockade of VLA-4/VCAM-1 interactions, careful scrutiny for the emergence of potentially fatal PML is required in patients receiving fingolimod.

The present data also demonstrated that fingolimod-phosphate restored the disruption of the barrier properties of the BBB induced by sera from both the RRMS-R and RRMS-S patients, although no significant differences regarding these effects were observed between the RRMS-R and RRMS-S groups. These data provide a theoretical basis indicating that treatment with fingolimod may be useful for ameliorating and suppressing MS relapse by repairing BBB breakdown.

No clear therapeutic options have been established in patients with SPMS to date. However, limited evidence has shown that fingolimod has therapeutic benefits in the progressive phase of MS [1,34–38]. Specifically, in one study, the progression of clinical disability over two years was significantly reduced by treatment with fingolimod compared to that observed in the placebo group, and the reduction in brain volume on MRI over one year was significantly smaller among the patients treated with fingolimod than in those treated with a placebo [38]. These findings were not observed in association with interferon-β treatment. These data are supported by the concept that fingolimod acts directly on neural and resident non-neural CNS cells, including astrocytes, oligodendrocytes and microglia, thereby inhibiting neurodegenerative processes. Interestingly, our present data showed that treatment with fingolimod-phosphate restores BBB damage, even after exposure to sera from SPMS patients. These findings thus provide a theoretical basis indicating that fingolimod-phosphate exerts neuroprotective effects in patients with SPMS by acting directly on BMECs, hence improving mild BBB disturbances, which may be associated with the progressive stage of the disease in SPMS patients.

In conclusion, the present findings demonstrated that pretreatment with fingolimod-phosphate enhances the barrier properties of the BBB by upregulating the claudin-5 expression and inhibiting the increased VCAM-1 levels in BMECs induced by MS sera. These results indicate that fingolimod may prevent the infiltration of autoaggressive lymphocytes into the CNS across the BBB via its direct effects on BMECs. The direct BBB-modulating effects of fingolimod may represent a possible novel venue for treating MS patients. Further studies are thus required to elucidate the overall mechanisms of action of fingolimod against BBB damage in the treatment of MS.
